# Round the Hospitals

**Published:** 1917-05-19

**Authors:** 


### ROUND THE HOSPITALS.

THE Central Committee's latest attempt to mislead and betray British nurses into a false position is a manifesto inviting each recipient to sign an enclosed petition to the Prime Minister hostile to the College. Nurses are asked to take this step on the following assertions:—

1. That the Central Committee consists of representatives of eight associations of nurses together with the B.M.A.

But at the dinner of the Guy's Hospital Nurses' League Miss Swift demonstrated clearly that there are in fact no really representative societies (or associations) which carry weight.

2. That the Central Committee represents some forty thousand professional men and women.

The facts are that after upwards of twenty years' propaganda its claim is to have recruited four thousand certificated nurses as members who are paying one shilling only, and this four thousand includes names on the College Register and some who may be disqualified for registration. Of whom do the remaining thirty-six thousand persons referred to consist? It cannot be of members of the British Medical Association, for they only number at present 20,443 members. Indeed, should the Committee include the whole of the registered practitioners whose names are on the Medical Register at present, they would only just suffice to provide the missing thirty-six thousand names. But for weeks, and indeed months, past the Central Committee has been regaled with the reiterated repudiation by its leader of "the intolerable assumption of authority over nurse-members" by medical officers and members of that much-abused profession. Who, then, in reality, are "the professional men and women" claimed as supporters of the party of "Make-Believe" and "Divide"? Names, please!

3. The Bill of the Central Committee is next referred to in four paragraphs, and the recipients of the circular are invited to approve "these principles" and sign an enclosed petition.

No mention is made of the Bill drawn up by the College of Nursing, Limited, "the principles" of which are entirely in accord with those of the Committee, the difference being only in method of procedure, the details of which can be ascertained by addressing a postcard to Miss Rundle, R.R.C., Secretary of the College of Nursing, 6 Vere Street, Cavendish Square, W. 1. We do not believe British Nurses are the fools and know-nothings that the Central Committee, by their circular, assume them to be. Before any nurse signs any document emanating from this Committee experience may have taught her, we hope, to hesitate long and learn all the facts before signing anything. At any rate, Mr. Lloyd George is not likely to be impressed by tactics such as those here exposed.

THE manifesto just referred to is interesting as an intimation that the party of "Divide" and "Make-Believe" are apparently pursuing botanical methods. It is a common practice amongst children to dig up a primrose root and re-plant it with its roots upwards in the belief that the yellow primrose will become pink when it next blooms. Children of persistence are apt to dig up the pink flower, should it appear, and again bury the plant with its root upwards in the belief that in the following year a fine polyanthus plant will appear. So, The All-Highest of the party of "Divide," while still retaining at the head of the official note-paper of the so-called "representative societies," the names of the chief office-holders who have not disagreed with and left T.A.H., has gradually had to surrender anonymity and to substitute names, whilst the former night sister is now established as Honorary Secretary of the Petition Sub-Committee. These are but a type of kaleidoscopic changes made in the process of manufacturing "representative societies" and their sub-sections, and they do not cost any money. They must give infinite amusement, as we hope, to the small group who claim to be "We, The Only British Nurses." They have become, at their birth, a source of ever-increasing amusement to the British Nursing World. Botany, however, does not apparently lend itself with a will to make-believes or pretentious humbug in any form.

---

THE nursing staffs of the hospitals of the Cape Hospital Board were seriously depleted during 1916, mainly through the demands of the military hospitals in South and East Africa and oversea, and the work of these institutions, in consequence, was carried on at a great disadvantage. At one of the hospitals it was found necessary to employ, temporarily, uncertificated ward assistants and to strengthen the organisation. The Board made arrangements, through their London agents and the South African Colonisation Society, to import four trained nurses whose travelling expenses were paid on condition that they would complete twelve months' service. In addition, the status of some of the smaller hospitals was raised to institutions recognised by the Colonial Medical Council, the sanction of that body having been given, in which a three years' (instead of a four years') course is required. Signs of discontent have recently been manifested amongst the nursing staffs of the institutions managed by the Cape Board, who then came to the conclusion that the enhanced monetary value of trained nursing under present conditions must be recognised, and all salaries (including matrons') were increased by 10 per cent. from January 1, 1917. The Union Government, too, have made some useful concessions to nurses in the matter of travelling expenses.

For "Food in Wartime," see p. 136; and "Matrons' and Sisters' Appointments" see p. 140.

